# Preharvest Methyl Jasmonate Treatment Increased the Antioxidant Activity and Glucosinolate Contents of Hydroponically Grown Pak Choi

**DOI:** 10.3390/antiox10010131

**Published:** 2021-01-18

**Authors:** Min Woo Baek, Han Ryul Choi, Tifsehit Solomon, Cheon Soon Jeong, Ok-Hwan Lee, Shimeles Tilahun

**Affiliations:** 1 Department of Horticulture, Kangwon National University, Chuncheon 24341, Korea; minwoo100@kangwon.ac.kr (M.W.B.); hanryul192@kangwon.ac.kr (H.R.C.); jeongcs@kangwon.ac.kr (C.S.J.); 2 Interdisciplinary Program in Smart Agriculture, Kangwon National University, Chuncheon 24341, Korea; 3 Department of Biology, Wollega University, Nekemte 395, Ethiopia; tifsehits@gmail.com; 4 Department of Food Science and Biotechnology, Kangwon National University, Chuncheon 24341, Korea; loh99@kangwon.ac.kr; 5 Agriculture and Life Science Research Institute, Kangwon National University, Chuncheon 24341, Korea; 6 Department of Horticulture and Plant Sciences, Jimma University, Jimma 378, Ethiopia

**Keywords:** antioxidant activity, DPPH, FRAP, ABTS, ORAC, glucosinolates, phenolics, flavonoids

## Abstract

Vertical hydroponics farming has emerged as an alternative solution to feed the continuously growing world population. Additionally, recent studies reported that the exogenous treatments of jasmonic acid influence the phytochemical composition of Brassicaceae. We conducted this study to determine the effect of preharvest methyl jasmonate (MeJA) treatment on the phytochemical composition and antioxidant activities of soil- and hydroponically grown pak choi. An aqueous solution of 0.5-mM MeJA was sprayed to saturation on the aerial plant part three days before harvest. The harvested pak choi was freeze-dried and then powdered to measure the antioxidant activity and the contents of chlorophylls (Chls), total phenolics and flavonoids, and glucosinolates (GSLs). The overall results revealed that pak choi grown in vertical hydroponics had higher total Chls and total phenolics than those grown in soil in the greenhouse, regardless of MeJA treatment. Nevertheless, the GSLs content and total flavonoids increased significantly due to MeJA treatment in both growing systems, and the highest values were recorded in hydroponically grown MeJA-treated pak choi. Similarly, the 2, 2-di-phenyl-1-picrylhydrazyl (DPPH) radical scavenging capacity, Trolox-equivalent antioxidant capacity (ABTS), oxygen radical absorbance capacity (ORAC), and ferric-reducing antioxidant power (FRAP) were highest in hydroponically grown MeJA-treated pak choi. Taken together, the preharvest foliar treatment of MeJA can be used to improve the phytochemical composition of pak choi grown in both growing systems. Interestingly, the results strongly support the use of MeJA treatment in the vertical hydroponics growing system compared to the conventional growing system in the soil. This indicates that supplementing the vertical hydroponic growing system with preharvest MeJA treatment could be the best option to improve both the yield per square meter and the quality of pak choi. Besides, MeJA-treated pak choi could be used as a value-added horticultural commodity, as its antioxidant activity increased after treatment. Moreover, after further studies, MeJA could also be applied to other Brassica vegetables to improve their GSL contents and antioxidant properties.

## 1. Introduction

Plant-based foods are rich sources of bioactive compounds that provide significant health benefits beyond essential nutrition. A minimum intake of 400 g of fruits and vegetables, excluding potatoes and other starchy tubers, is recommended per day for the prevention of chronic diseases [1,2]. Recent studies even indicate the importance of higher intakes than the daily general recommendations, and special attention has been paid towards edible crops that are rich in phytochemicals due to their antioxidant activity for the prevention of cancer, cardiovascular disease, and premature mortality [3,4,5]. Defending against oxidative and environmental stresses, including UV radiation, microbes, pathogens, and parasites, is the major function of phytochemicals. Besides, they are responsible for color and contribute to the taste of fruits and vegetables [6,7]. Phenolics, carotenoids, alkaloids, and organosulfur compounds are the major classes of phytochemicals [8]. According to previous reports, phytochemicals present in edible plants could be beneficial components for human health; the most widely studied activity of phytochemicals in humans is their antioxidant activity [6]. Antioxidants are substances that are stable enough to donate electrons to rampaging free radicals and neutralize them. This inhibition helps to prevent or delay cellular damage through their free radical scavenging property [9].

Based on the current reports, cruciferous vegetables are a good source of natural antioxidants and provide health-promoting phytochemicals such as carotenoids, vitamins, soluble sugars, fiber, phenolic compounds, and glucosinolates (GSLs) [10]. GSLs are characteristic bioactive compounds of Brassica vegetables, and various biotic and abiotic factors induce their biosynthesis [11,12,13]. GSLs are classified based on their precursor amino acid and the types of modification to the variable R group (aliphatic, aromatic, or indolic) [14]. The Brassicaceae/Cruciferae family includes the genus *Brassica*. The most popular, and economically important, worldwide species within the Brassica genera include *Brassica rapa* L, *Brassica oleracea* L., *Brassica juncea* L., and *Brassica napus* L. *Brassica rapa* L. includes vegetables such as pak choi, turnip, and Chinese cabbage, along with some oilseed and forage types [3].

Pak choi (*Brassica rapa* L. ssp. *Chinensis*) is a popularly consumed vegetable that shows an increasing trend of consumption mainly due to its comparatively mild flavor [12]. Pak choi is suitable to be cultivated in either the conventional methods in soil or in soilless hydroponics [15,16]. Vertical farming can be the best solution to feed the continuously growing world population, because it is unaffected by adverse weather conditions and provides reliable year-round production with a better use of space and minimum water usage [17]. Briggs et al. [15] reported over a three-fold increase in the yield of pak choi per square meter with a vertical hydroponic growing system.

Abiotic factors such as rainfall, solar radiation, and temperature and biotic factors like herbivore or pathogen attacks can influence the phytochemical composition of Brassicaceae [18]. Recent studies reported that the exogenous preharvest treatment of methyl jasmonate (MeJA) to mimic biotic stress also increased the total phenolics and antioxidant activity in kale [18]. MeJA is a certified safe compound for all food commodities when applied before harvest [19].

Generally, the growing system and exogenous treatments of elicitors like jasmonic acid are known to influence the quality of vegetables. Hence, we conducted this study to determine whether growing in soil or in vertical hydroponics with or without the application of MeJA was a better system for enhancing the Chl contents, GSL contents, total phenolics, and flavonoids, and antioxidant activities of pak choi.

## 2. Materials and Methods

### 2.1. Plant Material

**Figure 1:**
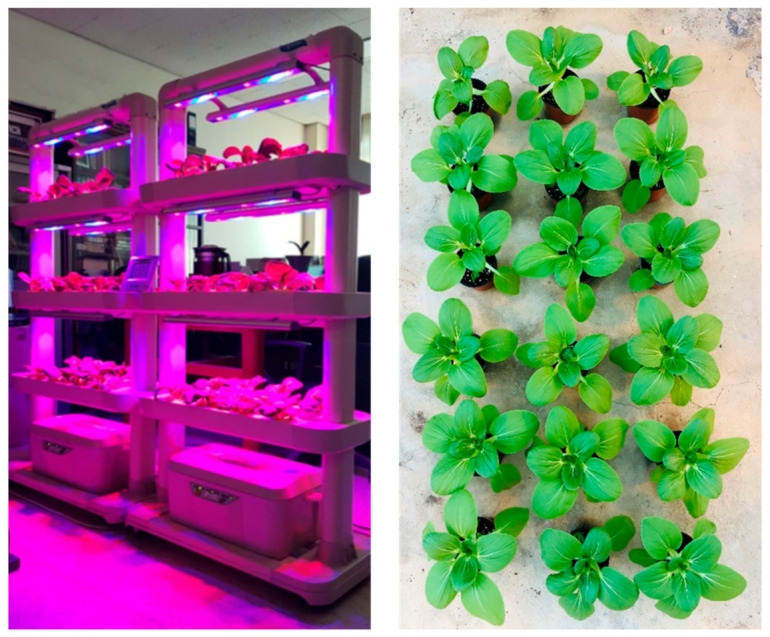
Transplanted pak choi plants in the fully equipped vertical growing bed with the hydroponic system (**left**) and in 1-L pots containing the soil mixture (**right**) at room condition and in the greenhouse, respectively.

Seeds of the pak choi cultivar “Baby bok choy” (Dongoh seed Co Ltd., Seoul, Korea) used in this study were germinated in 105 cell plant plug trays filled with horticulture nursery media (Punong Co Ltd., Gyeongju, Korea). Seedlings were grown in a greenhouse at the Kangwon National University under 23–25/19–21 °C day/night temperatures. Two weeks after germination, plants in the vegetative growth stage were transplanted to 1-L pots containing a soil mixture (Punong Co Ltd., Gyeongju, Korea) in the greenhouse and in a fully equipped 3-layer (8 growing points on each layer) vertical growing bed with the hydroponic system (Daesan precision neulpurenchae Co Ltd., Uiwang, Korea) at an ambient temperature and humidity (Figure 1). The electrical conductivity (EC) of the irrigation water in hydroponic system was adjusted at 2 deci Siemens per meter (ds m^−1^) at the beginning and increased up to 2.8 ds m^−1^ as the growth stage proceeded. Light was adjusted to 16/8 h light-dark cycles. Harvest maturity was attained after 30 days from transplant. When the plants were ready for harvest, an aqueous solution of 0.5-mM MeJA (Sigma-Aldrich, St. Louis, MO, USA) in 0.1% ethanol was sprayed on all aerial plant tissues to saturation three days prior to harvest, and the control plants were applied with a 0.1% ethanol solution [18]. The time of the harvest and treatment concentrations were based on previous study by Kim et al. [12].

Three days after spraying, eighteen plants were harvested for each treatment, with six pak choi plants in each of three biological replicates. All the samples were freeze-dried, grounded to fine powder, and filtered with 40-µm mesh and stored at −20 °C until extraction. Afterwards, all samples were extracted for 24 h on a rotating shaker (SI-600R, Medline Scientific, Oxfordshire, UK) with the speed of 140 rpm at room temperature by adding 80% ethanol 50 times the weight of the sample. After filtration using filter paper grade #2 (Advantec, Tokyo, Japan), a concentration of the extract was dried by vacuum evaporation at 40 °C in a rotary vacuum evaporator (N-1300, Eyela, Tokyo, Japan) and stored in a refrigerator prior to analysis.

### 2.2. Extraction and Quantification of Chls

Chls were extracted from the ethanol extracts of each treatment. A DMSO (dimethyl sulfoxide) chlorophyll extraction procedure was used, as described by Hiscox and Israelstam [20]. The readings were measured at 645 nm and 663 nm using a BioMate 3S UV-Visible spectrophotometer (Thermo Fisher Scientific, Waltham, MA, USA) against a DMSO blank. Subsequently, the total Chls, Chl a, and Chl b were calculated by the Arnon [21] equations as follows:

Chl a (mg g^−1^) peel fresh weight) = [(12.7 × A663) − (2.69 × A645)] × (V/1000 W)

Chl b (mg g^−1^) peel fresh weight) = [(22.9 × A645) − (4.68 × A663)] × (V/1000 W)

Total Chls = Chl a + Chl b

V = volume of the solvent, W = fresh weight of the extracted tissue.

### 2.3. GSLs Analysis

The extraction of desulfo-GSLs from the lyophilized 200-mg sample was performed according to Ku et al. [22]. Separation and quantification of desulfo-GSLs were performed according to the previously published methods by Han et al. [23] using a Dionex UltiMate 3000 ultra-high-performance liquid chromatography (UHPLC) system equipped with a column oven, pump, an auto-sampler, and a diode array detector (all Thermo Fisher Scientific, Waltham, MA, USA).

### 2.4. Total Phenolics Content

Folin-Ciocalteu assay [24] was used to measure the total phenolics content. Ethanolic extract (1 mL) containing 1 mg of the extract or standard was mixed with 1 mL of 10% Folin-Ciocalteu’s phenol reagent and 1 mL of 2% sodium carbonate solution. The absorbance was measured at 750 nm using a microplate reader (Spectramax i3, Molecular Devices, Sunnyvale, CA, USA) after incubation of the samples at ambient temperature for 90 min in dark. Comparison of the measurement to the calibration curve of gallic acid was made, and the results were expressed as milligrams of gallic acid equivalents (GAE) per gram of sample (mg GAE·g^−1^).

### 2.5. Total Flavonoids Content

A method described by Zhishen et al. [25] was used to measure the total flavonoids content. Water solution (0.5 mL) containing 0.5 mg of the extract was mixed with 1.5 mL of ethanol, 0.1 mL of 10% aluminum nitrite solution, 0.1 mL of 1-M potassium acetate solution, and 2.8-mL distilled water. The mixture was stirred and allowed to react for 30 min. Then, the absorbance was measured at 415 nm using a microplate reader (see Section 2.4). The measurements were compared to a rutin calibration curve, and the results were expressed as milligrams of rutin equivalents (RE) per gram of sample (mg RE·g^−1^).

### 2.6. Antioxidant Activities

#### 2.6.1. DPPH Radical Scavenging Activity

Sample solution (0.2 mL) containing different concentrations of the extract was added to a 0.8-mL ethanolic DPPH (0.4 mM) solution [26]. The solution was mixed and allowed to react at room temperature in the dark for 10 min. The absorbance was measured at 517 nm using a microplate reader (see Section 2.4). For the blank, distilled water was used instead of the sample. Then, the calculation for the radical scavenging activity was as follows:

DPPH radical scavenging activity (%) = [1 − (Absorbance of the sample/Absorbance of the blank)] × 100

#### 2.6.2. ABTS Radical Scavenging Activity

The 2, 2-azinobis-(3-ethylbenzothiazoline-6-sulfonic acid) (ABTS) radical scavenging activity of different concentrations of the extract was measured according to the modified method of Re et al. [27]. A stock solution of ABTS was dissolved in water to a 7.4-mM concentration. The cation (ABTS+) was produced by reacting the ABTS stock solution with 2.45-mM potassium persulfate and allowing the mixture to stand for 14 h at room temperature in the dark. The ABTS+ solution was diluted with ethanol to obtain an absorbance of 0.70 ± 0.02 at 750 nm. Then, 1.0 mL of diluted ABTS+ solution was added to 0.01 mL of sample, and the mixture was left at room temperature for 30 min in the dark. The absorbance was measured at 750 nm using a microplate reader (see Section 2.4). For the blank, distilled water was used instead of the sample. Then, the radical scavenging activity was calculated by the following equation:

ABTS radical scavenging activity (%) = [1 − (Absorbance of the sample/Absorbance of the blank)] × 100

#### 2.6.3. Ferric-Reducing Antioxidant Power (FRAP)

The FRAP assay for different concentrations of the extract was performed according to Kim et al. [28]. The FRAP reagent was prepared fresh daily by 300-mM acetate buffer (pH 3.6), a 10-mM 2, 4, 6-tri (2-pyridyl) -1, 3, 5-triazine (TPTZ) solution in 40-mM HCl, and a 20-mM FeCl3·6H2O solution in proportions of 10:1:1 (*v*/*v*), respectively. After preparation, the reagent was warmed to 37 °C in a water bath before use. Then, 0.05 mL of the sample was mixed with distilled water (0.15 mL) and the FRAP reagent (1.5 mL). The reaction mixture was incubated at 37 °C in a water bath for 4 min, and the absorbance was measured at 595 nm.

#### 2.6.4. Oxygen Radical Absorbance Capacity (ORAC)

The ORAC assay was performed by the method of Ou et al. [29]. Potassium sodium phosphate buffer (75 mM) was used to dilute the samples, and 0.025 mL of either the diluted samples or the diluted Trolox (0–10 μM), together with fluorescein (150 μL of 40 nM), was added into a black-walled 96-well plate. Then, 2, 2′-azobis (2-amidinopropane) dihydrochloride (AAPH) (25 μL of 18 mM) was preincubated for 15 min at 37 °C and transferred to each well, and the plate was immediately carried to the fluorescence microplate reader (see Section 2.4) to measure the fluorescence. Readings were recorded every 3 min for 90 min at 37 °C from the set analyzer at an excitation wavelength of 485 nm and an emission wavelength of 530 nm. Then, the results were calculated using a Trolox calibration curve and the area under the fluorescence decay curve. The result was expressed as Trolox equivalents in micromoles per milliliter.

Area under the curve (AUC) = 1 + f1/f0 + f2/f0 + f3/f0 +f4/f0 + f31/f0

where f0 is the initial fluorescence reading at 0 min, and fi is the fluorescence readings at every 3 min up to 90 min.

### 2.7. Statistical Analysis

The experiment was conducted in a completely randomized design, and data were subjected to an analysis of variance (ANOVA) to determine the significant differences between treatments at *p* < 0.05 using SAS statistical software (SAS/STAT ^®^ 9.1; SAS Institute Inc., Cary, NC, USA). Duncan’s multiple range test was performed to observe differences between the treatment means. Principal component analysis (PCA) was analyzed using XLSTA version 2015.1 (Addinsoft Inc., 244 Fifth Avenue, Suite E100, New York, NY, USA).

## 3. Results and Discussion

### 3.1. Effect of MeJA Treatment on Chl Contents of Pak Choi Grown on Soil and in a Hydroponic System

**Figure 2:**
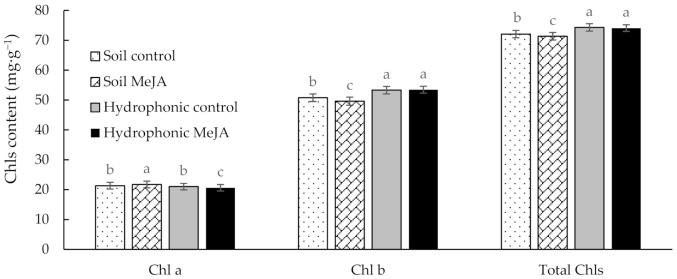
Chlorophyll (Chl) a, Chl b, and total Chl contents of pak choi grown in soil or hydroponics with or without the treatment of methyl jasmonate (MeJA). Different letters on the bars indicate a significant difference (*p* < 0.05) between the treatments. The vertical bars represent the standard error of the mean (*n* = 3).

The natural bioactive compounds in vegetables include vitamins, minerals, antioxidants, and pigments (chlorophylls and carotenoids) [30]. In addition to capturing light for photosynthesis in the living plants, pigments exhibit color appearances that influence consumer preferences by indicating maturity, quality, and freshness [31]. Esposito and Giugliano [32] reported the association of a greater intake of green leafy vegetables with a 14% reduction in the risk of type 2 diabetes. Chls are the most prominent natural pigments in green vegetables that exhibited considerable antioxidant activity [33,34]. Hence, the Chl content could be a good indicator of quality in pak choi. In this study, the preharvest MeJA treatment had no significant difference in the Chl content of pak choi grown in hydroponics. However, MeJA-treated soil-grown pak choi reduced the Chl content as compared to the control. The total Chl contents of MeJA-treated pak choi were 74.07 and 71.35 mg g^−1^ in the hydroponics and soil, respectively. Similarly, the total Chl contents of the control were 74.30 and 72.07 mg g^−1^ in the hydroponics and soil, respectively. The overall results revealed that pak choi grown in hydroponics had higher total Chls than those grown in soil in the greenhouse (Figure 2). This could be explained by the exposure of the plants to excess light in the greenhouse. Chls are usually synthesized and photo-oxidized in the presence of light. Nevertheless, excess light can cause greater degradation and, consequently, a reduction in the levels of total Chls. On the other hand, under a deficit or optimum set light conditions (like in the hydroponics system in the present study), the plants could increase their photosynthetic pigments for the efficient use of the available light. Similar to the present study, Rezai et al. [35] also reported an increase in Chl contents of sage plants with a decreasing light intensity. Thus, growing pak choi in a vertical hydroponics system could be beneficial in terms of the total Chls, regardless of MeJA treatment.

### 3.2. Effects of the MeJA Treatment on GSL Contents of Pak Choi Grown in Soil and in a Hydroponic System

**Figure 3:**
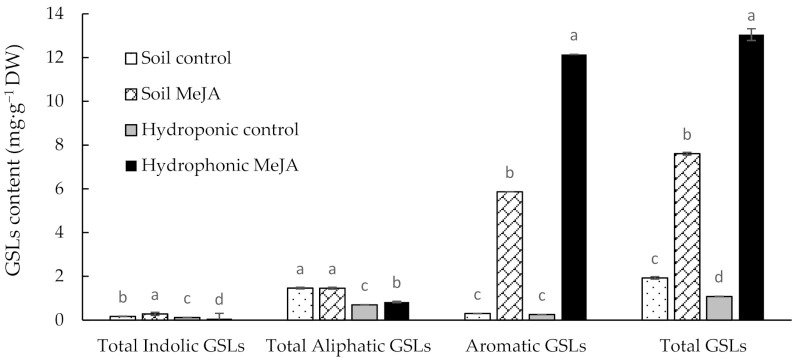
Glucosinolate contents of pak choi grown in soil or hydroponics with or without the treatment of MeJA. Different letters on the bars indicate a significant difference (*p* < 0.05) between the treatments. The vertical bars represent the standard error of the mean (*n* = 3).

As shown in Figure 3, it appears that, in the absence of MeJA treatment, soil cultivation could lead to higher total GSLs than hydroponic cultivation. However, the GSL concentration increased significantly in response to the preharvest MeJA treatment in both the soil and hydroponics growing conditions. In the hydroponics, the total GSLs were 13.05 and 1.08 mg·g^−1^ dry weight (DW) in the treated and control, respectively. Similarly, the total GSLs in the soil cultivation were 7.61, and 1.93 mg·g^−1^ DW for the treated and control, respectively (Figure 3). A total of 12 GSLs were detected, with aromatic gluconasturtiin (GNT) as the predominant GSL, representing 77% and 93% of the total GSL contents in MeJA-treated soil- and hydroponically grown pak choi, respectively (Table 1). The treatment increased the total GSLs by four- and 12-fold in soil and the hydroponics, respectively, as compared to the control (Figure 3 and Table 1). Interestingly, the treatment of preharvest MeJA enhanced two-fold total GSL contents in the hydroponics growing condition as compared to the corresponding MeJA-treated soil-grown pak choi. Similar to the present study, Van Dam et al. [36] reported 1.5–3 times increase in the total shoot GSL levels of Brassica oleracea and Brassica nigra with jasmonic acid treatment. In addition, Ku et al. [22] reported a significant increase of gluconasturtiin (56%), glucobrassicin (98%), and neoglucobrassicin (150%) concentrations in the apical leaf tissue of Brassica oleracea genotypes with foliar 250-mM MeJA treatment four days prior to harvest. Smetanska et al. [37] also reported an increase in the total amount of GSLs in turnip plants and plant parts after 30 d of treatment with salicylic acid (SA) and MeJA to the roots through the nutrient solution. In line with the present study, 2-phenylethyl glucosinolate (gluconasturtiin) was the only aromatic GSL in the turnips, and it increased in 3.8-fold after the MeJA application [37].

### 3.3. Effects of MeJA Treatment on the Total Phenolic and Flavonoid Contents of Pak Choi Grown in Soil and in the Hydroponic System

**Figure 4:**
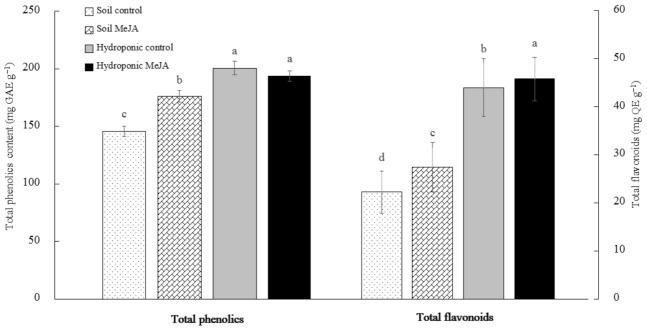
Total phenolic and flavonoid contents of pak choi grown in soil or hydroponics with or without the treatment of MeJA. Different letters on the bars indicate a significant difference (*p* < 0.05) between the treatments. The vertical bars represent the standard error of the mean (*n* = 3).

The total phenolics content of preharvest MeJA-treated pak choi was significantly higher than the control in the soil-growing systems. However, hydroponically grown MeJA-treated pak choi was statistically similar to the control (Figure 3). This indicates that the preharvest MeJA treatment could either increase or not affect the total phenolics content, depending on the growing conditions. Our results were in agreement with the previous work by Kim et al. [38], who reported a significant increase of the total phenolics within 48 h after 1-mM MeJA treatment on radish sprouts. In our study, the preharvest 0.5-mM MeJA treatment also increased the total flavonoid contents of both soil- and hydroponically grown pak choi (Figure 4). Similar to our results, an exogenous application of a low MeJA dose (10 μM MeJA) was shown to promote an accumulation of phenolic compounds, particularly a 31% increase in flavonoids in seven-day-old broccoli sprouts [39].

### 3.4. Effects of the MeJA Treatment on the Antioxidant Activities of Pak Choi Grown in Soil and in the Hydroponic System

The overall results revealed the significant (*p* < 0.05) increase of antioxidant activities in response to the preharvest MeJA treatment in both the soil and hydroponics growing conditions (Table 2). All the antioxidant activity determination methods used in this study, the DPPH radical scavenging capacity, ABTS, FRAP, and ORAC, showed the same trends with the total phenolics and flavonoids (Figure 3), regardless of the sample concentrations. The antioxidant activities showed an increasing trend in all methods as the concentrations of the extracts increased (Table 2). The highest scavenging capacity was recorded from MeJA-treated hydroponically grown pak choi, followed by either the hydroponically grown control or MeJA-treated soil-grown. The lowest scavenging capacity was recorded from the soil-grown control (Table 2). Polyphenols are major plant compounds with antioxidant activity [40]. Sokół-Łętowska et al. [41] also stated that polyphenols are believed to have the ideal chemical structures for free radical scavenging. In addition to polyphenols, the higher antioxidant capacity of MeJA-treated hydroponically grown pak choi in the present study could be attributed to the significantly higher content of Chls and total GSLs (Figure 2 and Figure 3). In green vegetables, Chls are the most prominent natural pigments that exhibit considerable antioxidant activity [33,34]. Additionally, the bioactivity of GSLs and their hydrolysis products have been reported [42,43]. Thus, growing pak choi in a vertical hydroponics system with MeJA treatment could be beneficial in terms of improving the antioxidant activity as compared to the soil-grown control.

### 3.5. Principal Component Analysis

**Figure 5:**
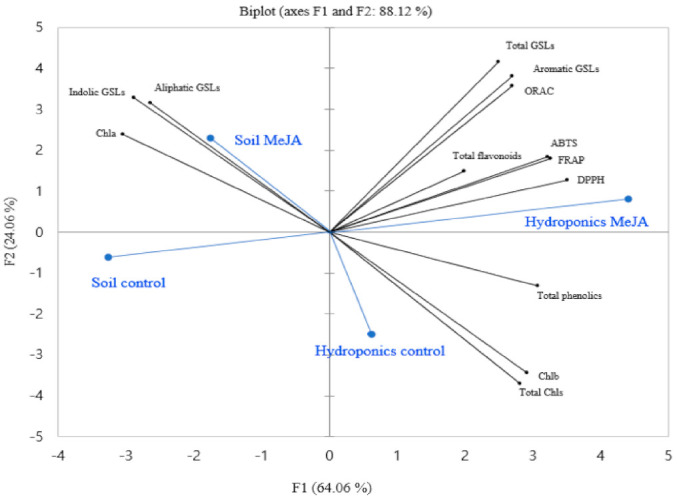
Biplot of the principal component analysis of the observed parameters of pak grown in soil or hydroponics with or without the treatment of MeJA. ● Observed parameters ● Treatments.

The difference between the treatments and the observed parameters that largely affect the spatial distribution could be better explained through the principal component analysis. This method can also describe the correlation of the observed parameters [44]. The PCA of the observed parameters in this study is presented in Figure 5. The contents of the Chls and GSLs, total phenolics and flavonoids, and antioxidant activities were used as variables to perform the PCA. The treatments were separated based on the above parameters, and factor 1 (F1) and factor 2 (F2) comprised about 88.12% of the total variance. F1 showed about 64.06% of the total variance and positively correlated to the total GSLs; aromatic GSLs; antioxidant activities (ORAC, ABTS, FRAP, and DPPH); total phenolics and flavonoids; Chl b; and total Chls. As shown in Figure 5, the above parameters were also higher in hydroponically grown MeJA-treated pak choi as compared to the controls. The F2 accounted for about 24.06% of the total variance and mainly represented variances in the indolic GSLs, aliphatic GSLs, and Chl a. Taken together, the preharvest MeJA treatment showed a great impact on the measured parameters, mainly in the hydroponic growing system (Figure 5). Hence, supplementing the vertical hydroponic growing system with a foliar application of 0.5-mM MeJA aqueous solution prior to harvest could be the best option to improve the antioxidant properties and GSLs of pak choi.

## 4. Conclusions

In this study, we compared the antioxidant activities, the contents of the Chls, total phenolics, total flavonoids, and GSLs of soil- and hydroponically grown pak choi after a foliar application of 0.5-mM MeJA aqueous solution three days prior to harvest. To the best of our knowledge, there has been no experiment conducted to compare the effects of a preharvest MeJA treatment on pak choi in different growing systems. The overall results revealed that pak choi grown in vertical hydroponics had higher total Chls and total phenolics than those grown in soil in the greenhouse, regardless of the MeJA treatment. Nevertheless, significant increases in the concentrations of the GSLs, flavonoids, and antioxidant activities were observed in MeJA-treated pak choi grown in both the soil and hydroponics systems compared to the untreated control. Interestingly, the results strongly support the use of a MeJA treatment in the recently emerging vertical hydroponics growing system as compared to the conventional growing system in the soil. This indicates that supplementing the vertical hydroponic growing system with a preharvest MeJA treatment could be the best option to improve both the yield per square meter and the quality of the pak choi. Besides, MeJA-treated pak choi could be used as a value-added horticultural commodity, as its antioxidant activity increased after treatment. Moreover, after further studies, MeJA could also be applied to other Brassica vegetables to improve their GSL contents and antioxidant properties.

## Figures and Tables

**Table 1:** Individual glucosinolate contents of pak choi (*Brassica rapa* L. ssp. *Chinensis*) grown in soil or hydroponics with or without the treatment of methyl jasmonate (MeJA).

Treatments		GSLs Content (µg/g DW)														
		Aliphatic								Indolic					Aromatic	Total GSLs
		PGT	GRA	GIB	SIN	GAS	GNP	GBN	Total	GBS	4HGB	4MTGB	NGB	Total	GNT	
Soil	Control	33.9a	89.9b	4.8a	4.9ab	20.0bc	1190.3a	119.6b	1463.4a	70.1b	7.8b	74.1a	17.9a	169.9b	299.2c	1932.5c
	MeJA	29.5a	102.7a	2.1c	4.4b	22.1b	1121.8b	174.6a	1457.2a	200.2a	1.7c	63.2b	18.3a	283.3a	5866.8b	7607.4b
Hydroponics	Control	19.6b	94.5b	2.7b	5.3a	16.5c	488.2d	78.1c	704.9c	56.0c	10.1a	46.7c	10.5b	123.3c	255.4c	1083.5d
	MeJA	17.6b	72.8c	1.5d	3.0c	34.5a	576.7c	127.0b	833.0b	30.7d	0.5c	35.8d	3.3c	70.3d	12145.1a	13048.4a

PGT, progoitrin; GRA, glucoraphanin; GIB, glucoiberin; SIN, sinigrin; GAS, glucoalyssin; GNP, gluconapin; GBN, glucobrassicanapin; GBS, glucobrassicin; 4HGB, 4-hydroxyglucobrassicin; 4MTGB, 4-methoxyglucobrassicin; NGB, neoglucobrassicin; and GNT, gluconasturtiin. The glucosinolate content is presented as the mean of three replicates. Different letters in the same column indicate a significant difference (*p* < 0.05) between the treatments.

**Table 2:** The DPPH (2, 2-di-phenyl-1-picrylhydrazyl) radical scavenging capacity, Trolox-equivalent antioxidant capacity (ABTS), ferric-reducing antioxidant power (FRAP), and oxygen radical absorbance capacity (ORAC) of pak choi (*Brassica rapa* L. ssp. *Chinensis*) grown in soil or hydroponics with or without the treatment of MeJA.

Parameters	Sample Concentration (mg·mL^−1^)	Treatments			
		Soil		Hydroponics	
		Control	MeJA	Control	MeJA
DPPH (%)	1.25	39.06 ± 0.69d	41.30 ± 0.38c	42.52 ± 0.06b	47.75 ± 0.29a
	2.5	51.26 ± 0.25c	57.09 ± 0.46b	56.91 ± 0.08b	68.83 ± 0.33a
	5	73.78 ± 0.60d	82.40 ± 0.67c	84.71 ± 0.17b	92.84 ± 0.14a
ABTS (%)	1.25	12.88 ± 0.24c	14.39 ± 0.54b	13.34 ± 0.70c	18.11 ± 0.15a
	2.5	20.24 ± 0.47b	19.92 ± 1.1b	20.33 ± 0.81b	27.80 ± 0.12a
	5	32.20 ± 0.53d	33.95 ± 0.21b	33.07 ± 0.17c	49.05 ± 0.32a
FRAP (Absorbance)	1.25	0.18 ± 0.002d	0.23 ± 0.002c	0.24 ± 0.006b	0.27 ± 0.001a
	2.5	0.36d ± 0.001d	0.47 ± 0.009c	0.49 ± 0.009b	0.55 ± 0.007a
	5	0.52 ± 0.01d	0.71 ± 0.007b	0.66 ± 0.008c	0.84 ± 0.013a
ORAC (µm Trolox)	0.01	10.26 ± 2.26ab	10.34 ± 1.55ab	4.45 ± 2.18b	11.66 ± 0.74a
	0.05	25.24 ± 0.52c	37.09 ± 0.35b	23.78 ± 0.10d	65.45 ± 0.59a
	0.1	45.10 ± 0.11c	65.20 ± 0.21b	41.35 ± 0.08d	99.00 ± 1.12a

Results are presented as the mean ± SD from triplicate independent values. Means with different letters within the same row are significantly different at *p* < 0.05.
